# Supplementary material for: Turning Seashell Waste into Electrically Conductive Particles
Source: Int J Mol Sci. 2022 Jun 29;23(13):7256. doi: 10.3390/ijms23137256 (PMC9267058; doi:10.3390/ijms23137256)
Supplement: Supplementary file 1 [file ijms-23-07256-s001.zip › ijms-1737724-supplementary.pdf]

Supplementary Materials

Turning Seashell Waste into Electrically Conductive Particles

Stefanie Gärtner [1], Angelina Graf [1], Carla Triunfo [2], Davide Laurenzi [3], Stefan M. Schupp [4], Gabriele Maoloni [3], Giuseppe Falini [2] and Helmut Cölfen [1,*]

[1] Department of Chemistry, Physical Chemistry, University of Konstanz, Universitätsstrasse 10, Box 714, D-78457 Konstanz, Germany; stefanie.gaertner@uni-konstanz.de (S.G.); angelina.graf@uni-konstanz.de (A.G.)
2 Dipartimento di Chimica "Giacomo Ciamician", Alma Mater Studiorum Università di Bologna, via F. Selmi 2, 40126 Bologna, Italy; carla.triunfo2@unibo.it (C.T.); giuseppe.falini@unibo.it (G.F.)
[3] Finproject S.p.A., Plant Ascoli Piceno, Via Enrico Mattei, 1 - Zona Ind.le Campolungo, 63100 Ascoli Piceno, Italy; d.laurenzi@finproject.com (D.L.); g.maoloni@finproject.com (G.M.)
[4] Department of Physics, University of Konstanz, Universitätsstraße 10, Box 714, D-78457 Konstanz, Germany; stefan.schupp@uni-konstanz.de
* Correspondence: helmut.coelfen@uni-konstanz.de; Tel.: +49 (0) 7531 88-4063 (Office), +49 (0) 7531 88-2027 (Secretary Ms. Köst), Fax +49 (0) 7531 88-3139

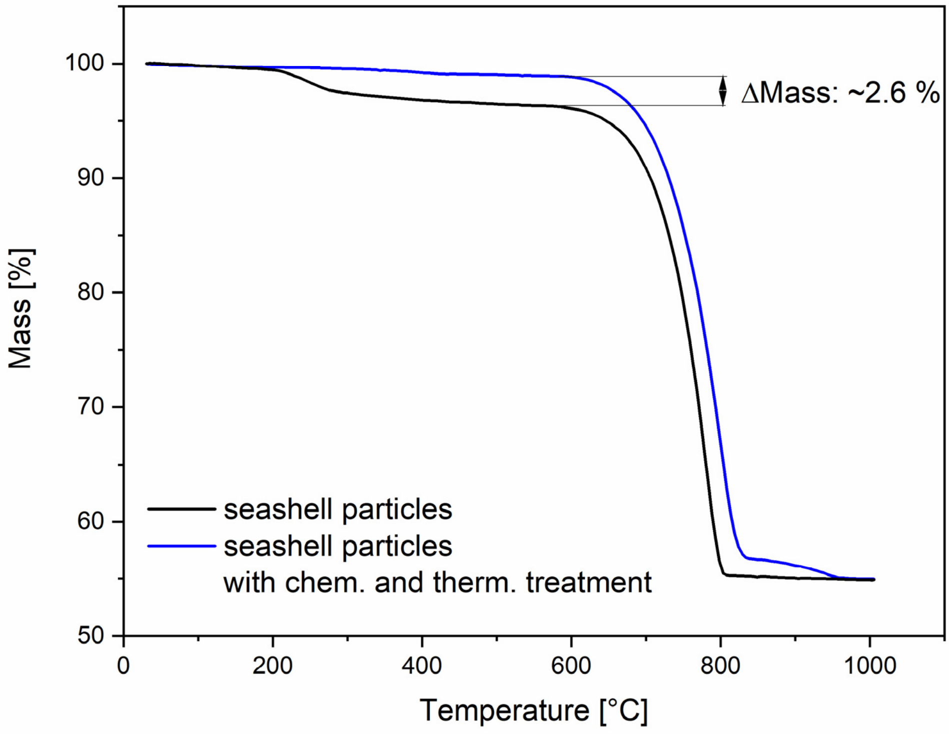

Figure S1. TGA image of seashell particles (black) andseashell particles that have been chemically and thermally treated to remove the organic content (blue).

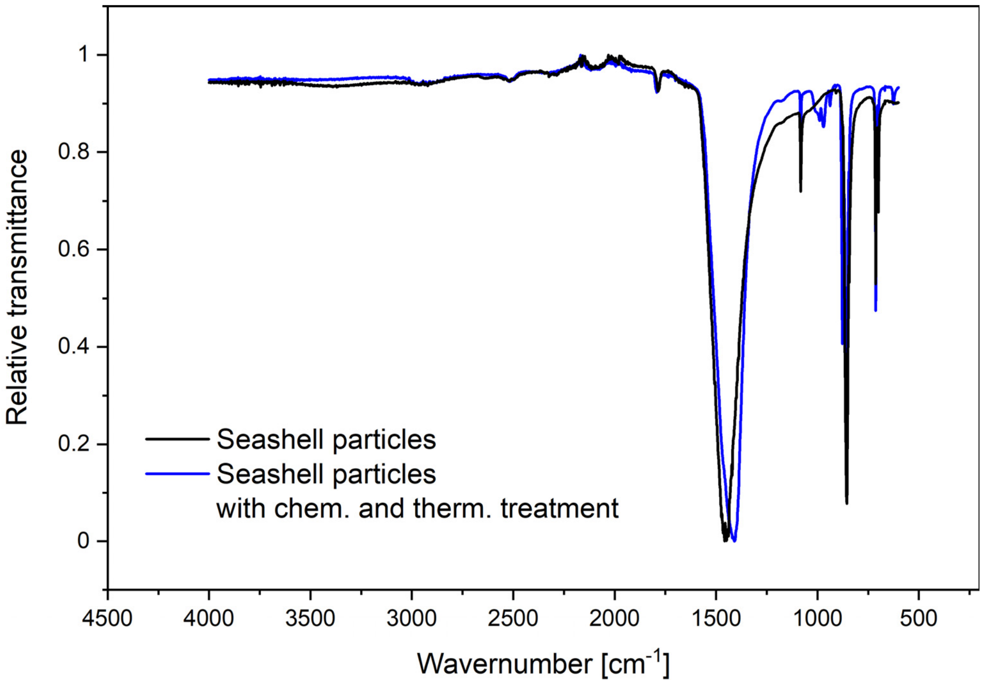

Figure S2. ATR-IR image of seashell particles (black) and seashell particles that have been chemically and thermally treated to remove the organic content (blue). The characteristic peaks at 1410 cm^{-1}, 1082 cm^{-1}, 857 cm^{-1}, 712 cm^{-1}, and 700 cm^{-1} are consistent with those of aragonite.

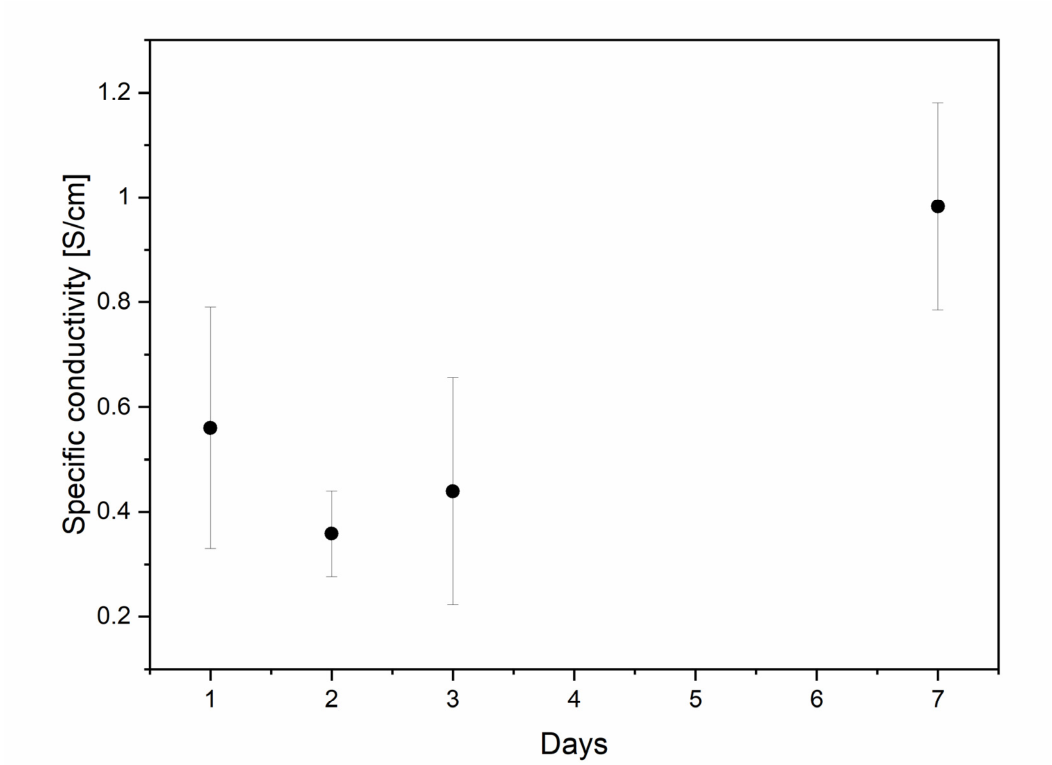

Figure S3. Specific conductivity of PPy/$CaCO_3$ composites (PPy/$CaCO_3$ 3 material) with different doping durations (1, 2, 3, 7 days).

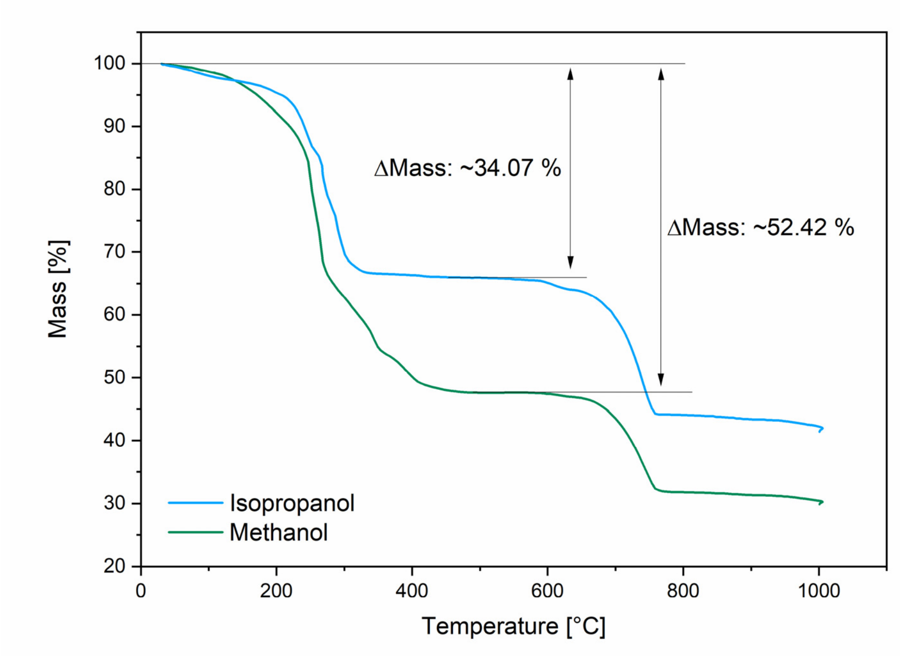

Figure S4. Comparing the polymer amount, which was measured with TGA, of PPy/$CaCO_3$ composite materials (PPy/$CaCO_3$ 5 and 6) which were formed in different solvents: Isopropanol and methanol.

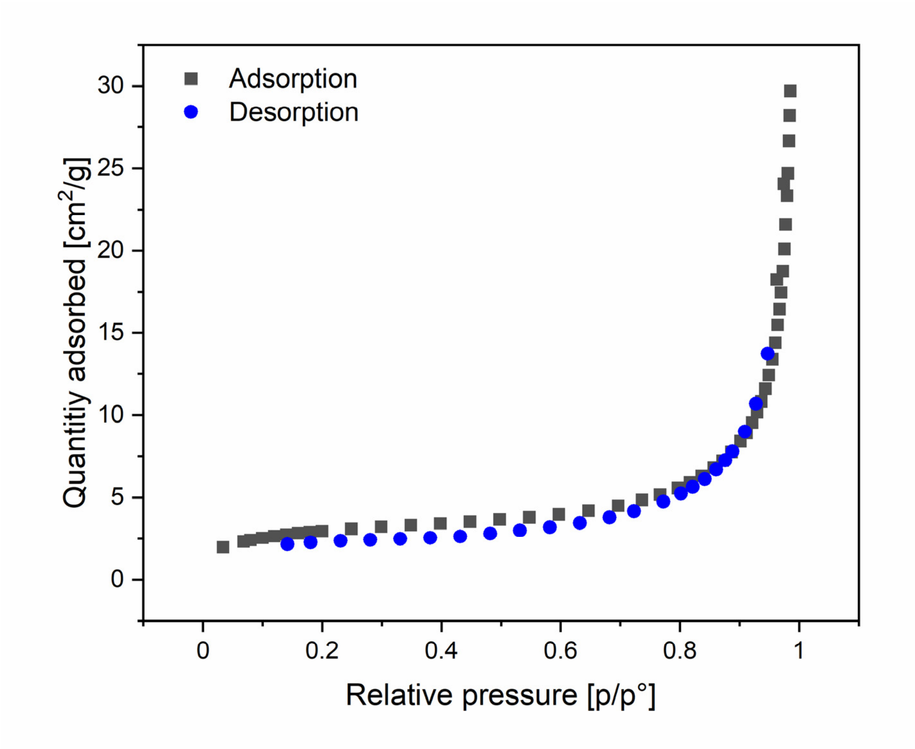

Figure S5. Nitrogen adsoprtion and desorption isotherm for PPy/$CaCO_3$ composite

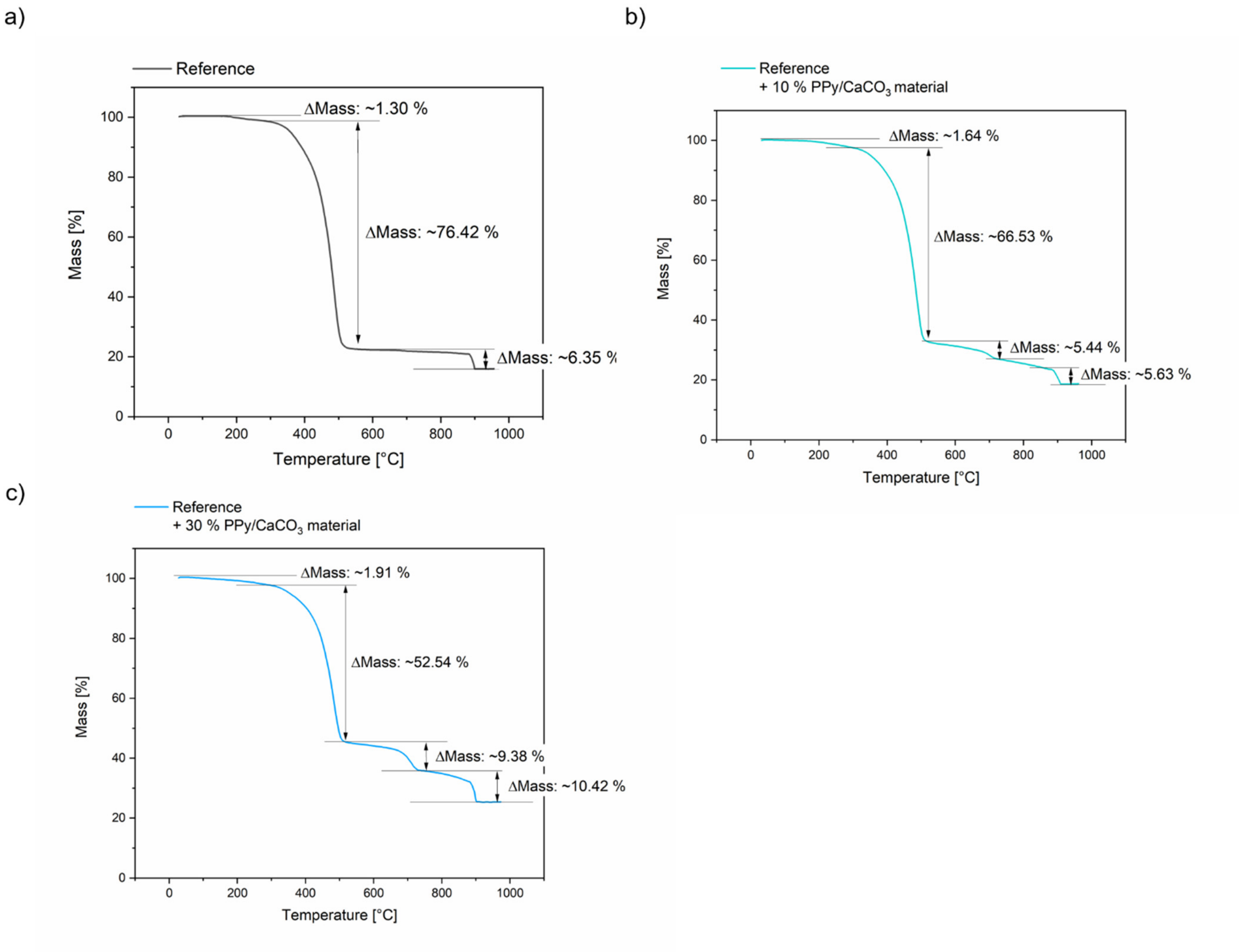

Figure S6. Thermogravimetric analysis of the reference sample Levirex® LX B/518 (**a**), PPy/$CaCO_3$ particles (30 wt % and 10 wt%) added to sole material. PPy/$CaCO_3$ particles (30 % (**b**) and 10 % (**c**)) molded in sole material Levirex® LX B/282.

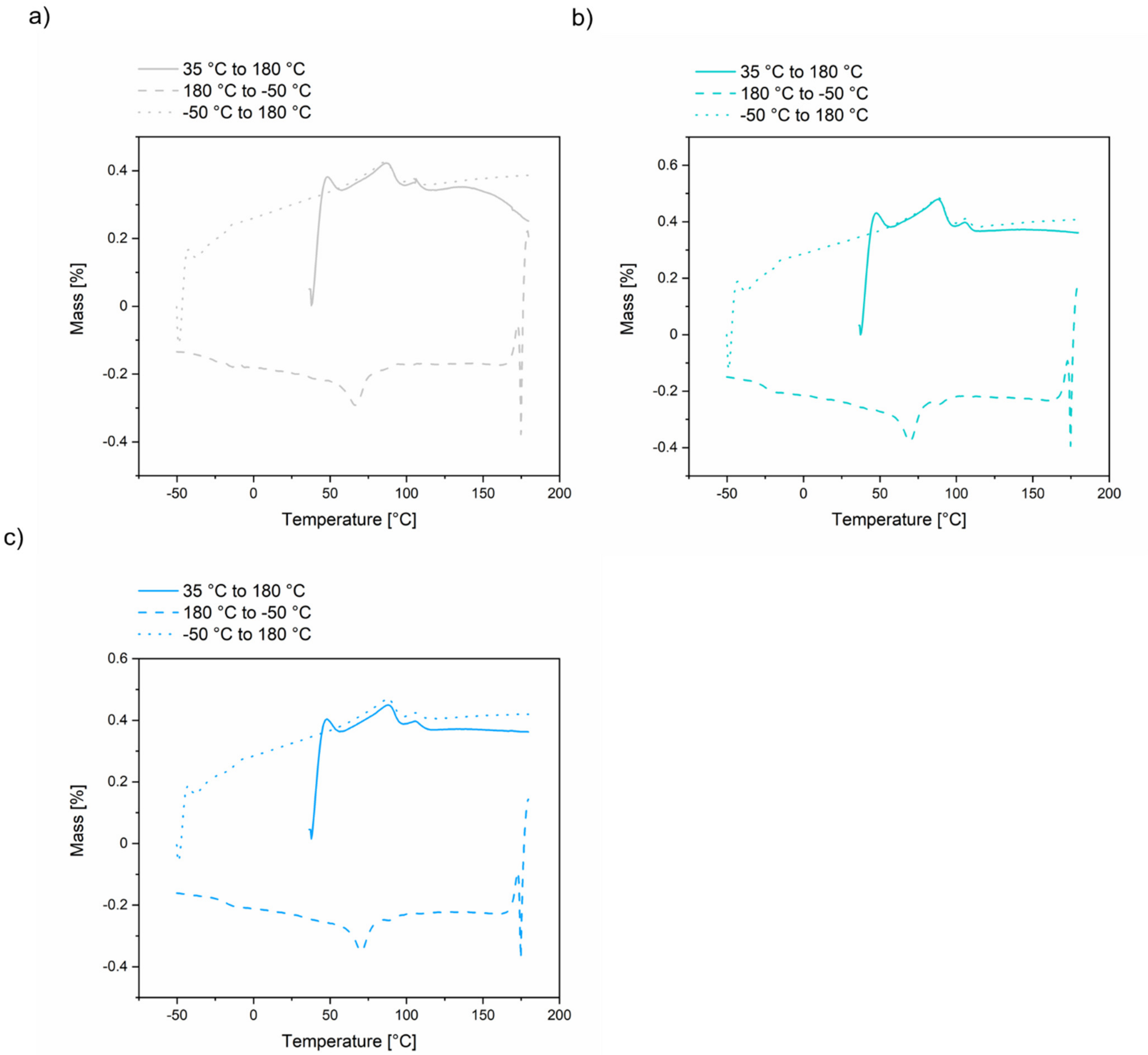

Figure S7. Differential scanning calorimetry of the reference sample Levirex® LX B/518 (**a**), PPy/$CaCO_3$ particles (30 wt % and 10 wt%) added to sole material. PPy/$CaCO_3$ particles (30 % (**b**) and 10 % (**c**)) molded in sole material Levirex® LX B/282.

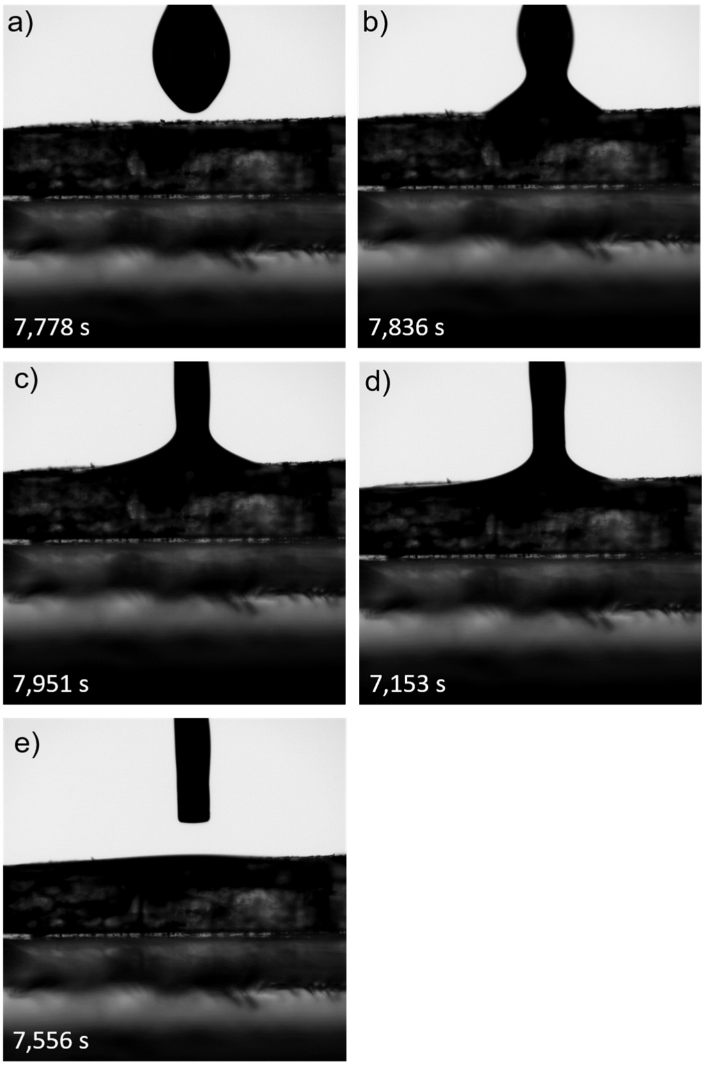

Figure S8. Contact angle measurement of pyrrole/methanol (2:1) mixture on a calcite surface.
